# Dutch fog: On the observed spatio‐temporal variability of fog in the Netherlands

**DOI:** 10.1002/qj.3597

**Published:** 2019-08-02

**Authors:** Jonathan G. Izett, Bas J. H. van de Wiel, Peter Baas, J. Antoon van Hooft, Ruben B. Schulte

**Affiliations:** ^1^ Department of Geoscience and Remote Sensing Delft University of Technology Delft The Netherlands; ^2^ Meteorology and Air Quality Group Wageningen University and Research Wageningen The Netherlands; ^3^ Netherlands National Institute for Public Health and the Environment (RIVM) Bilthoven The Netherlands

**Keywords:** climatology, fog, land use, regional variability, weather

## Abstract

The Netherlands is characterized by highly variable land use within a small area, and a strong influence of the North Sea on national climate. Devoid of significant topography, it is an excellent location for assessing the relative influence of various factors on fog occurrence in the absence of terrain effects. Using observations from a dense network of weather stations throughout the country, the climatology of fog in the Netherlands is assessed over a period of 45 years. On a national scale, interannual variability is linked to changes in synoptic pressure‐gradient forcing. Within the country, a comprehensive in‐depth analysis of regional differences between fog occurrence is made, together with an assessment of local physical factors which could bias fog formation in one location over another. Regional variability is shown to be strongly related to the mesoscale influences of urbanization and the North Sea. In fact, some locations experience over twice as much fog as others. From this finding, a simple index is presented, which combines the water and urban fraction surrounding a station. This “Regionally Weighted Index” (*RWI*) is able to accurately sort the stations according to their relative fogginess. Its practical use is encouraged for assessing a given site's climatological favourability, even when *in situ* meteorological observations are unavailable.

## 1 INTRODUCTION

**Figure 1:**
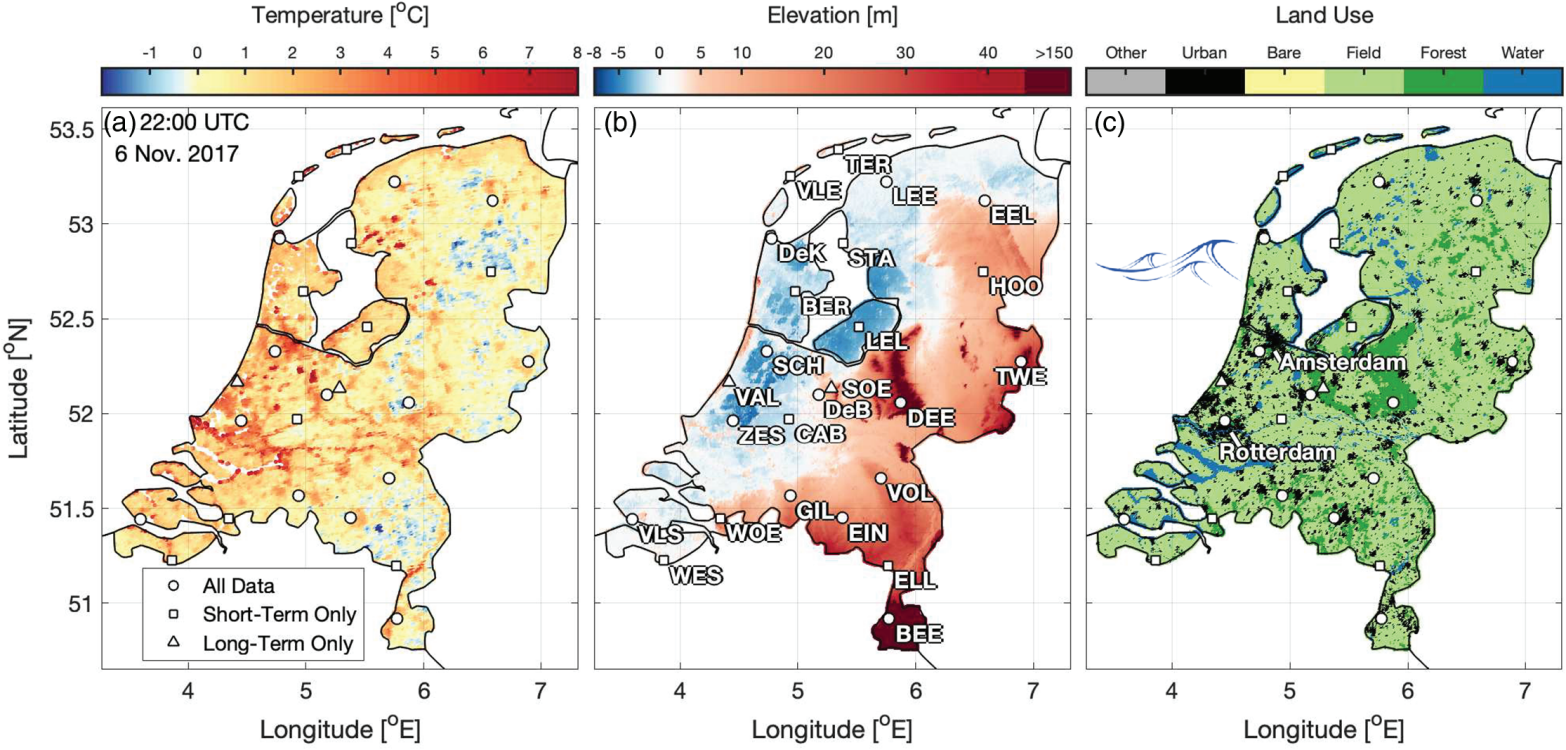
Heterogeneity in the Netherlands. (a) MODIS‐observed nocturnal land surface temperature under clear‐sky conditions on 6 November 2017 (Wan *et al.* 2015), (b) AHN2 surface elevation, and (c) ESA‐CCI land use (Hollmann *et al.* 2013). The North Sea lies to the northwest, while IJsselmeer is the enclosed lake northeast of Amsterdam. The weather stations used in this work are indicated by white points. Details of the stations can be found in Section 2.1 and Table 1, with a browsable map at https://jonathan‐izett‐research.weebly.com/dutch‐fog.html; accessed 3 July 2019

Fog is a hazard that impacts all modes of transport, yet it remains challenging to predict its occurrence using numerical models. In part, this is because of the need to capture both the large‐scale processes, e.g. the evolution of synoptic weather systems, as well as to accurately describe the local small‐scale processes, such as surface fluxes and microphysics (Gultepe *et al*. 2007; Steeneveld *et al*. 2015). In this paper we seek to better understand the influence of various factors on the spatio‐temporal variability of fog by analyzing climatologies of fog observations from a dense network of weather stations spread throughout the Netherlands. Two datasets are used, one long‐term dataset spanning 45 years, and a short‐term dataset that is 6 years long (Section 2.1). While the Netherlands is a relatively small, flat country, land use and population density vary significantly. Combined with the influence of the North Sea, the varied landscape can lead to significantly different localized weather conditions. This is particularly apparent in the absence of other external forcing, such as during clear‐sky nights with weak‐wind conditions, when the near‐surface temperature can vary by several degrees, even over short distances (e.g. Figure 1a). Correspondingly, the frequency and type of fog events are expected to be highly

variable throughout the country as well. We seek to identify factors on a range of spatial scales – from mesoscale to synoptic – that influence Dutch fog occurrence regionally and on interannual time scales. The aim of this work is twofold: (a) to report on the observed spatio‐temporal variability of fog in the Netherlands, and (b) to relate the observed variability to external influencing factors that can be used to better identify when and where fog is most likely to occur.

**Table 1:** Overview of stations in the analysis, including elevation, distance to the ocean (*D*_o_), data coverage, and physical setting

			Lon.	Lat.	Elev.	*D*_o_	Long‐term	Short‐term	
Station			(°)	(°)	(amsl)	(km)	data	data	Setting
1.	ASS	Assendelft	4.73	52.48	–2.0	9.2	—	2012–2018	Polder/Agricultural
2.	BEE	Beek	5.77	50.92	112.7	173.7	1955–2000	2012–2018	Rolling terrain; forest nearby
3.	BER	Berkhout	4.98	52.64	–2.4	23.8	—	2012–2018	Flat polder; grassland/arable
4.	CAB	Cabauw	4.93	51.97	–0.7	44.6	—	2012–2018	Grass polder
5.	DEE	Deelen	5.87	52.06	45.2	98.7	1955–2000	2012–2018	Slightly sloped; shrub/forest
6.	DeB	De Bilt	5.18	52.10	1.9	53.5	1955–2000	2012–2018	Half open grass/arable with buildings
7.	DeK	De Kooy	4.78	52.92	0.6	3.9	1955–2000	2012–2018	Coastal polder
8.	EEL	Eelde	6.58	53.12	3.2	35.3	1955–2000	2012–2018	Flat/open grassland
9.	EIN	Eindhoven	5.38	51.45	20.7	107.3	1955–2000	2012–2018	Half open mixed vegetation
10.	ELL	Ell	5.76	51.20	30.0	146.2	–	2012–2018	Grass
11.	GIL	Gilze‐Rijen	4.94	51.57	11.9	75.6	1955–2000	2012–2018	Half open grass/arable
12.	HOO	Hoogeveen	6.57	52.75	15.8	75.8	—	2012–2018	Open arable
13.	LEE	Leeuwarden	5.75	53.22	0.3	12.3	1955–2000	2012–2018	Flat polder; mainly grass
14.	LEL	Lelystad	5.52	52.46	–4.4	62.4	—	2012–2018	Grass polder
15.	MUI	Muiden	5.09	52.34	–5.6	37.9	—	2012–2018	Polder/Agricultural
16.	NWK	Nieuwkoop	4.76	52.15	–1.2	26.0	—	2012–2018	Polder/Agricultural
17.	NWV	Nieuw Vennep	4.65	52.25	–4.9	13.6	—	2012–2018	Polder/Agricultural
18.	SCH	Schiphol	4.74	52.33	–4.2	15.5	1955–2000	2012–2018	Mixed polder
19.	SOE	Soesterberg	5.28	52.13	14.0	58.2	1955–2000	—	Forest
20.	STA	Stavoren	5.38	52.90	–1.3	18.1	—	2012–2018	Open grassland
21.	TER	Hoorn Terschelling	5.35	53.39	0.7	2.9	—	2012–2018	Grass polder
22.	TWE	Twente	6.89	52.27	33.0	145.5	1955–2000	2012–2018	Slightly sloped/rolling; mixed surface
23.	VAL	Valkenburg	4.42	52.16	–0.2	3.9	1955–2000	—	Flat/open grassland
24.	VLE	Vlieland	4.94	53.25	1.7	0.5	—	2012–2018	Dune
25.	VLS	Vlissingen	3.60	51.44	8.0	<0.1	1955–2000	2012–2018	Urban/coastal
26.	VOL	Volkel	5.71	51.66	19.9	109.3	1955–2000	2012–2018	Half open mixed grass/arable and forest
27.	WES	Westdorpe	3.86	51.22	1.7	31.3	—	2012–2018	Open polder; grass/arable
28.	WOE	Woensdrecht	4.34	51.45	15.0	44.9	—	2012–2018	Forested
29.	ZES	Zestienhoven	4.45	51.96	–5.1	19.1	1956–2000	2012–2018	Grass polder

Locations can be seen in Figure 1. More information is contained in the interactive map at https://jonathan‐izett‐research.weebly.com/dutch‐fog.html

Previous observational studies of fog have focused primarily on single‐site observations. For example, Dutch fog has been studied extensively through observations at the Cabauw site (e.g. Duynkerke, 1991; Duynkerke, 1999; Izett *et al*. 2018; Izett *et al*. 2019) located in the centre of the Netherlands. Likewise, observational campaigns such as at the ParisFog site in France, (e.g. Haeffelin *et al*. 2010), at the CIBA site in Spain (e.g. Román‐Cascón *et al*. 2016), and the FRAM project in Canada (Gultepe and Milbrandt, 2007) look primarily at local fog occurrence at independent locations. Often, the measurement sites are at or near airports due to the need for accurate real‐time information about current visibility conditions. While this is important from a practical standpoint, the local setting of an airport – with buildings, runways, and often near major urban centres – is not a representative landscape; a regional study with multiple diverse measurement locations is therefore desirable.

However, regional studies are much less common. This is likely due to the difficulty in obtaining dense, compatible observations across large areas. In this regard, the contribution from Tardif and Rasmussen (2007) provides an excellent example in the literature of an investigation of regional fog variability. They analyzed 20 years of fog observations in and around the New York City region on the northeastern coast of the United States. With 17 stations in an area approximately half the size of the Netherlands, their study region features complex terrain, with deep river valleys (such as the Hudson) and dense urban centres (including Manhattan). Overall, they concluded that fog occurs most frequently at coastal, rural and suburban stations, with the least fog in urban settings. However, topographic effects, while significant in determining a local fog climatology, potentially obscure other underlying influences related to such properties as land use.

Further regional studies include work by Bendix (2002), who investigated the regional occurrence of fog and low stratus in Germany (and surrounding areas). Using 10 years of satellite imagery, Bendix found significant variability throughout the region, both in terms of fog occurrence, as well as fog type. However, the study was limited to observations during satellite overpasses free of high cloud, and was unable to distinguish between fog and low cloud. More recently, Egli *et al*. (2019, 2017) presented an analysis of satellite‐derived fog occurrence over Europe, focusing on the continental‐scale patterns of fog distribution, which are heavily influenced by topography. In a much smaller area, Cereceda *et al*. (2002) studied the occurrence of fog at a handful of sites in the Atacama desert in Chile, showing the coastal influence on fog type. Finally, Price *et al*. (2018) conducted a series of observations and simulations in England during the 18‐month Local and Non‐Local Fog Experiment (LANFEX). Within the LANFEX regions of interest, small‐scale topographic features were important in determining local fog occurrence (particularly the deepening of fog layers) through their influence on the turbulent properties of the stable boundary layer. However, the relatively small areal extent of the region, and similarly short‐term extent of the observations, make it difficult to assess the wider regional and climatic influence of other factors. All studies are heavily influenced by topography.

In contrast to the other study regions, and many regions around the world, the Netherlands provides a unique setting to study regional influences on fog in what is a largely topographically uniform country. Figure 1b shows the surface elevation from the second Actueel Hoogtebestand Nederland dataset (AHN2; “Current Height of the Netherlands”; http://www.ahn.nl/index.html; accessed 3 July 2019). Except in the southeast, much of the Netherlands is flat, low‐lying terrain. As a result, the influence of various factors can be investigated without the additional complexity, and potential obscuration, of topographic effects. At the same time, the small scale of the country (a land area of less than 35,000 km^2^), large variability in land use (Figure 1c from the European Space Agency's Climate Change Initiative database, ESA‐CCI; Hollmann *et al*. 2013), and extensive network of observations allow for a highly detailed look at different influences in a range of settings.

Not only is regional variability important to investigate, but temporal variability as well. Boers *et al*. (2015) noted that, on average, the occurrence of fog in the Netherlands has decreased significantly since the mid‐1950s. They largely attributed the underlying long‐term trend to a combination of decreased aerosol concentrations, offset by increased aerosol hygroscopicity. However, their study used the average observations of five stations spread throughout the country – as opposed to the individual trends at each station – which hides any potential regional variability. Likewise, they did not investigate interannual variability of the signal, which is not monotonic in time. Similarly, while Tardif and Rasmussen (2007) used 20 years of observational data, they did not investigate the interannual and long‐term trends in the data. Here we will therefore assess the long‐term trend and interannual variability in fog occurrence in order to gain a better understanding of climatic variability.

Several factors are expected to influence the overall Dutch fog climate on regional and national scales. For example, the Dutch landscape is characterized by large agricultural regions, interspersed with dense cities (Figure 1c). The Randstad area, for instance, is the densely populated region in the west of the country containing the cities of Amsterdam, The Hague, Utrecht, and Rotterdam. Nestled within the Randstad region is the “Groene Hart” (Green Heart) of the Netherlands, an agricultural region dominated by the classical polder landscape. Urban environments typically experience higher nocturnal temperatures due to the heat island effect, which has been shown to impact the local fog climate at various sites (e.g. Bendix, 1994; Sachweh and Koepke, 1995; Sachweh and Koepke, 1997; Steeneveld *et al*. 2011). At the same time, the possibility of a “wind island effect” (Droste *et al*. 2018) and the enhanced roughness of cities has implications for the downwind turbulent characteristics, potentially impacting the favourability of a site for fog formation. However, the presence of urban features may not be entirely detrimental to the formation of fog, with increased aerosols and moisture as the result of anthropogenic emissions (Hage, 1972). Similarly, the presence of the North Sea and the IJsselmeer (the large freshwater lake in the north of the country) have a significant impact on local weather conditions, through such effects as sea‐breeze circulation, which can alter aerosol concentration (Arrillaga *et al*. 2018) and local temperatures, as well as the modulation of diurnal and seasonal temperature cycles through the increased heat capacity of the water. At the same time, the North Sea also brings with it a thermal memory in the form of the Gulf Stream circulation, making the coastal waters warmer than they would otherwise be without the transatlantic transport of heat from the tropical and Equatorial regions (Palter, 2015). Further, its location in northwestern Europe means that the Netherlands is influenced by large‐scale teleconnections, such as the North Atlantic Oscillation (NAO), and possibly even the El Niño/Southern Oscillation (ENSO) in the tropical Pacific, which can have significant impact on northern European weather (e.g. Toniazzo and Scaife, 2006; Hirschi and Sinha, 2007; Riaz *et al*. 2017; King *et al*. 2018). However, the extent to which these factors influence the occurrence of fog on interannual and spatial scales remains unclear.

Section 2 describes the data used in this work as well as the methods of analysis. Following in Section 3, the fog climatologies are presented, along with investigations into the observed spatio‐temporal variability of fog, including the development of an index to describe mesoscale variability in fog occurrence. A discussion of the results follows in Section 4, with recommendations for the practical application of the results.

## 2 OBSERVATIONAL DATA AND METHODS

### 2.1 Meteorological data

The meteorological data used in this study are obtained from the land‐based weather station network maintained by the Royal Netherlands Meteorological Institute (KNMI). All stations conform to World Meteorological Organization standards (e.g. over grass; WMO, 2014). Table 1 provides an overview of the stations and their data coverage, while the weather station locations can be seen in Figure 1, as well as in an interactive Google map (embedded at https://jonathanizett-research.weebly.com/dutch-fog.html).

On average, the stations are separated from their nearest neighbour by 30 km. The smallest separation between neighbouring stations is 7 km, and the maximum separation 50 km. Weather station coverage is densest in the Randstad region. The majority of the weather stations are located in grass/cropland settings, with many of the stations at (former) airfields. Two main exceptions to this are the Vlissingen station (VLS; located within an urban setting on a peninsula) and Vlieland (VLE; on one of the Wadden Sea islands where the weather station is in a dune environment). Likewise, most of the stations are located in fairly flat terrain within a few metres of sea level, and with negligible slopes. The Beek station (BEE; located at Maastricht airport in the southeast of the country) is the only major exception to this, being over 100 m above mean sea level (amsl), with more complex terrain surrounding the station. Other stations with local elevation elements (though on a much smaller scale) are, for example, Twente (TWE) and Deelen (DEE).

Two observational records are used; one long‐term dataset, and one short term. The bulk of the analysis was conducted using the long‐term dataset of hourly observations dating back to 19551
http://projects.knmi.nl/klimatologie/metadata; accessed 3 July 2019; also used by Boers *et al*. 2015
. Hourly observations of visibility, temperature, relative humidity, atmospheric pressure, cloud cover, and wind speed/direction are used. Other variables are available, but are not considered in this analysis. All stations operated continuously throughout the day, with no consistent observational gaps that would be associated with infrequent sampling. A range of observational methods were employed to determine visibility, including the use of dedicated observers and later transmissometers. While the observation method changed, there are no obvious discontinuities in the data before the year 2000, with variability also similar between different stations. After the year 2000 there appear to be some inconsistencies in the records as the instruments were again changed in the early 2000s. Therefore, we perform the long‐term analysis only up to, and including, the year 2000. At the same time, we restrict our analysis to stations that have complete records (≥90% of data in a given year) for at least 40 years. Overall, the long‐term dataset consists of 15 stations with complete records.

We also use data from a short‐term dataset of 10‐min averaged observations from 27 automatic weather stations (AWS) for the years 2012–2017 in order to assess the occurrence of individual fog events. Thirteen of the 15 long‐term stations are also in the short‐term dataset. The AWS record meteorological optical range (MOR), which is an objective measure of the visibility (WMO, 2014). While the absolute measurements may differ from the “historical” observations of visibility, our results are not influenced as we avoid making any direct comparisons between the two datasets. For simplicity, we also use the term “visibility” throughout the rest of the paper to refer to both the long‐term visibility observations and MOR.

### 2.2 Assessing fog occurrence

Fog is defined as conditions where the observed visibility is less than 1 km (NOAA, 2005). We compare the occurrence of fog at different stations by first assessing the total count of observed fog, *n*_fog_ (i.e. the number of observations where the visibility is at or below 1 km) in a given month or year. To account for variations in month length, observational record, and the possibility of missing data, the total count is converted to a fog fraction, *F*_fog_, which is *n*_fog_, divided by the number of valid observations over the comparison period, *n*_obs_:

(1) $$F_{\text{fog}}=\frac{n_{\text{fog}}}{n_{\text{obs}}}.$$

Whether calculating the monthly or annual value, we restrict ourselves to periods where at least 90% of the observations are valid, to avoid gaps in the record influencing our results. We define the mean of *F*_fog_ over all stations as the “Dutch mean”. In order to remove general temporal trends (e.g. long‐term decreases, or interannual variability), we divide *F*_fog_ at each station by the Dutch Mean. Taking the mean of this relative value over the entire data record gives a station's “relative fogginess”, *RF*, over the long‐term period.

The number of fog events is also diagnosed for the short‐term data. As in Tardif and Rasmussen (2007), a fog event is identified when conditions are foggy (here using the visibility threshold of ≤1 km) for at least 50 min out of one hour (at least five out of six consecutive 10‐min observations). Two events are then deemed independent when separated by at least two hours as in Román‐Cascón *et al*. (2016). While there are several types of fog (each defined by their formation process), we restrict the bulk of our analysis to the occurrence of fog *in general*, regardless of the type of fog. The exception is that we investigate the relative occurrence of radiation fog – formed on weak‐wind, clear‐sky nights under strong nocturnal cooling due to the net imbalance of long‐wave radiation – and other fog types. This is because the formation of radiation fog (as opposed to, e.g. advection fog) is primarily dependent on local cooling processes indicative of the underlying substrate and immediate surroundings. Such properties vary on regional scales, which should lead to regional variability in the occurrence of radiation fog. For the 21 out of 27 short‐term stations with cloud data available, a simplified version of the Tardif and Rasmussen (2007) algorithm is used to classify fog events as radiation fog based on the conditions before onset. An event is classified as radiation fog if, in the hour prior to onset, cloud cover is less than 10%, 10‐m wind speed is below 2.5 m/s, and the air temperature decreased. No precipitation or ceiling data are available, so they are ignored. If the above criteria are not met, then an event is classified as “other”. The use of such simple criteria can – and likely does – result in some mis‐classification of events, including missing some radiation events, or classifying “other” events incorrectly as radiation fog. It also neglects the possibility of combination types, such as advection‐radiation fog, which is likely represented in both categories of “radiation” and “other” used here. However, we assume that the mis‐classification works in both directions, while at the same time primarily assessing fog in general. As such, the possibility of mis‐classification does not significantly affect the results presented herein.

## 3 RESULTS

### 3.1 Underlying meteorological conditions at the AWS

Before assessing the occurrence of fog at the weather stations, we first present the observed underlying meteorological conditions at the stations in order to highlight synoptic similarity and regional variability.

**Figure 2:**
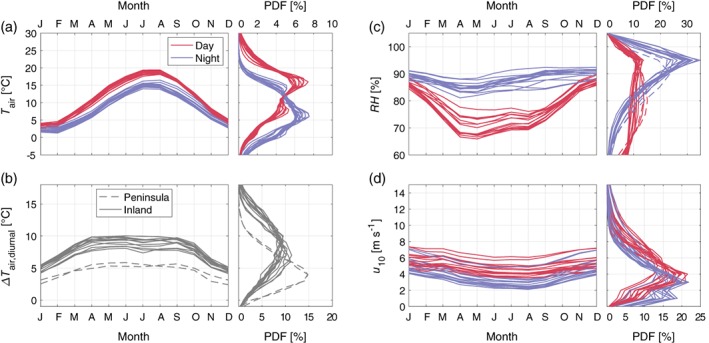
Monthly distributions of mean values and probability density functions of observed meteorological variables at the long‐term stations: (a) daytime/nocturnal air temperature, (b) diurnal temperature difference (daytime maximum minus night‐time minimum) with the peninsular stations of Vlissingen (VLS) and De Kooy (DeK) shown in dashed lines, (c) daytime/nocturnal relative humidity, and (d) daytime/nocturnal wind speed [Colour figure can be viewed at wileyonlinelibrary.com]

Overall, daytime and nocturnal air temperatures follow fairly uniform seasonal cycles at the weather stations (Figure 2a). Day and night are determined according to local sunrise/sunset times, excluding the hour on either side of sunrise/sunset. Peak temperatures are observed in July and August, although coastal stations have a delayed peak (September), coinciding with the North Sea surface temperature (Figure S1a in the Supporting Information). The probability density function (PDF) of temperatures is also fairly consistent between stations. However, the differences in observed temperature between stations are magnified when the mean diurnal cycle (daytime maximum temperature minus night‐time minimum temperature) is assessed (Figure 2b). The stations at Vlissingen (VLS) and De Kooy (DeK), shown with dashed lines, exhibit much smaller diurnal temperature ranges, with a mean day–night difference of only 5 °C. Both stations are located on peninsulas within 1 km of the North Sea (Vlissingen) or the IJsselmeer (De Kooy). Conversely, some stations have diurnal cycles of 10 °C throughout the year. All stations have a fairly uniform diurnal cycle throughout the spring to autumn, with the weakest diurnal variability in winter.

Related to temperature, the seasonal cycle of daytime relative humidity is pronounced for all stations (Figure 2c), with mean daytime relative humidity around 85% in winter, and as low as 70% in spring and summer. In general, nocturnal relative humidity is more uniform throughout the year, with slightly elevated relative humidity in autumn and winter. The lowest nocturnal relative humidity values, and highest daytime values, are observed at the coastal stations (reduced diurnal variability). While the ocean provides abundant moisture, it appears the reduced cooling near the coasts means the nocturnal relative humidity does not reach the same high values typically observed at the inland stations.

Seasonally, mean wind speeds at all stations vary by approximately 3–5 m/s, with stronger mean winds in winter than in summer (Figure 2d). However, there is a large spread in observed wind speeds between stations, with inland stations experiencing much weaker winds overall. Vlissingen (VLS) has the highest wind speed observations, with mean values above 6 m/s throughout the year.

### 3.2 Observed fog climatology

**Figure 3:**
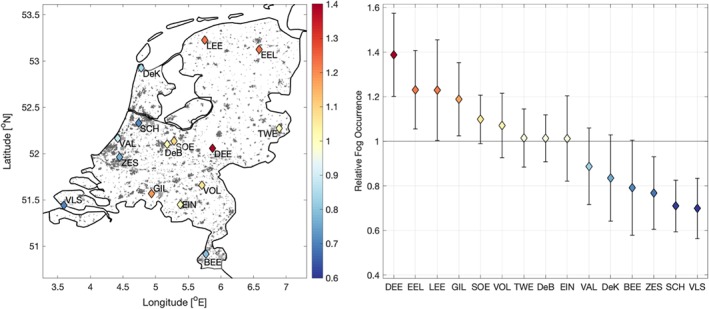
Relative occurrence of fog (compared to the Dutch mean) at each station between 1955 and 2000. The errorbars indicate one standard deviation (± 1 sd)

Here we assess the overall fog climatology at each station. Compared to the Dutch mean, relative fogginess at the long‐term stations ranges from 0.75 to 1.4 (Figure 3; Section 2.2 gives definitions). In an absolute sense, the factor of 2 difference corresponds to over 200 hourly observations of fog per year. It should be noted that similar interstation variability is observed when different metrics are used to assess fog occurrence, such as the total number of days on which fog is observed, or the number of non‐consecutive fog observations.

Overall, stations in the centre and northeast of the country exhibit higher fog occurrence than stations in the south and west, particularly along the coast of the North Sea. The fewest observations of fog were recorded at Vlissingen (VLS), which is located in an urban setting on a peninsula. Low fog occurrence is also observed at Beek (BEE; located in the most complex terrain of all stations in the southeast of the country) and, notably, Schiphol International Airport (SCH). Deelen (DEE) – located within a forest clearing next to the largest national park in the Netherlands – was observed to experience the most fog. On this, we note that analysis of the short‐term dataset agrees with the long‐term analysis: similar spatial patterns are observed, with less fog near the coast. Vlissingen, Beek, and Schiphol again have the lowest observed fog occurrence.

Interesting to note is the difference in fog occurrence, even over short distances. For example, the De Bilt (DeB) and Soesterberg (SOE) stations are located within 7 km of each other. However in most years, Soesterberg is 10% foggier than De Bilt in the long‐term record. This is likely due to the more urbanized setting of the De Bilt station, which is located at the KNMI headquarters just outside the city of Utrecht, whereas Soesterberg is a more rural location that was a military air base until 2008.

More than 50% of all of the observed fog events (in the short‐term dataset) were classified as radiation fog events. However, the actual fraction of radiative events varies according to station (Figure S2). For example, coastal stations experience only 10% radiation fog events, with the inland rural station at Ell having the greatest proportion of radiation fog events (82%). Overall, the frequency of radiation fog events increases inland. However, while the number and type of events varies, the character of events (i.e. onset time, event duration, mean visibility) are similar across all stations (Figure S3). When a fog event is observed at one station, an event is generally also observed at one or more other stations within a few hours (>90*%* of all events; not shown). However, fog events at Beek (BEE; hilly station in the south, located furthest from any other station) occur more often in isolation from events at the other stations (20% of all events at Beek occur in isolation).

**Figure 4:**
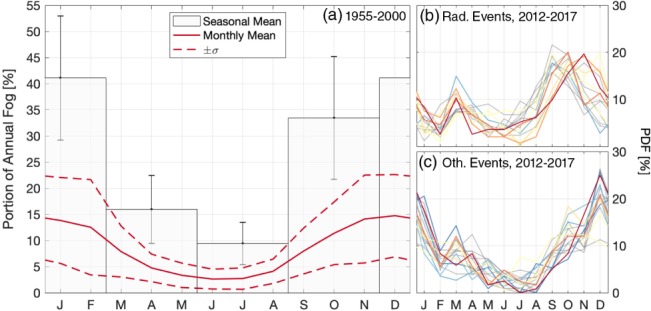
Mean fog occurrence throughout the year. (a) Seasonal and monthly fog occurrence between 1955 and 2000, averaged for all stations in the Netherlands. Monthly occurrence of (b) radiative, and (c) non‐radiative fog events between 2012 and 2017. The colours are the same as in Figure 3

Seasonally, on average fog occurs most frequently in the autumn and winter months, accounting for 75% of the total annual fog (Figure 4a). All stations exhibit a nearly identical annual distribution to the Dutch mean, with the most fog occurring in late autumn and winter, and the least fog in summer (Figure S1b). That being said, the peninsular stations of De Kooy (DeK) and Vlissingen (VLS) exhibit the largest seasonal amplitudes, with less than 2% of the annual fog occurring in July and August, and greater than 17% of the annual fog in each of the winter months. Their seasonal signals also lag the mean signal by one month, corresponding to the seasonal sea surface temperature of the North Sea, which reaches its maximum in September. Conversely, the stations with the most fog overall have the most uniform seasonal distribution, with the summer months having at least 10% of the annual fog. This points to local favourability of the sites as they are able to form fog in otherwise less favourable conditions (i.e. shorter nights). Likewise, the general seasonal pattern is also observed in the short‐term data, with radiative events occurring most frequently in autumn, and uniformly throughout the rest of the year (Figure 4b). The other fog types, including advection fog, occur almost exclusively in winter (Figure 4c) when the land is considerably cooler than the ocean. This is what drives the strong seasonal cycle at the coastal stations where “other” fog is more common.

**Figure 5:**
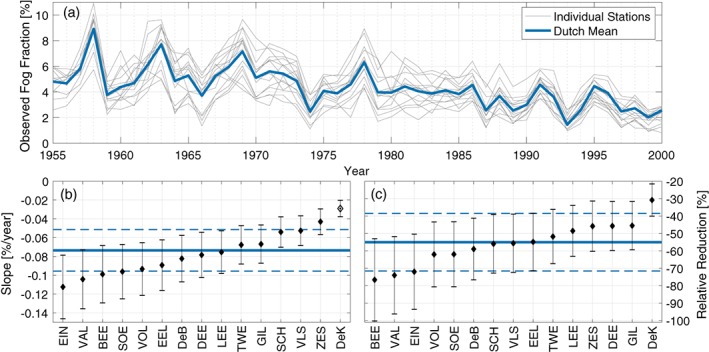
Annual occurrence of fog from 1955 to 2000 at each station, and the mean of all stations. (a) Observed fog fraction (*F*_fog_), (b) long‐term trend in fog fraction (slope) obtained through linear regression, and (c) relative reduction in fog over 40 years, compared to the 1955 value. Note that the trend at De Kooy (DeK) is not statistically different from 0 [Colour figure can be viewed at wileyonlinelibrary.com]

Boers *et al*. (2015) showed that the occurrence of fog in the Netherlands has decreased significantly since the 1950s. However, they looked at the trend based on the mean annual occurrence of five stations. Figure 5 shows that the long‐term trend dating back to 1955 is significantly different at different stations. Fitting a linear regression to the long‐term annual fog occurrence between 1955 and 2000, the mean trend (with 95% confidence interval) of all stations is –0.07±0.02% per year (Figure 5b). While this is negligible on an annual time‐scale, over five decades this amounts to a total reduction of 3.3±1.0% (roughly half of the 1955 value; Figure 5c). The station with the most rapid decline in fog occurrence between 1955 and 2000 is Eindhoven (EIN) with a slope of −0.11±0.03% per year, amounting to a total reduction between 1955 and 2000 of 72% of the original 1955 value! Conversely, the trend at De kooy (DeK) is statistically insignificant.

### 3.3 Factors influencing the interannual variability of fog occurrence

The observed fog occurrence in the Netherlands over the past several decades is punctuated by large interannual variability (Figure 5a). In many cases, the interannual variability is far greater than the interstation variability, and long‐term decline in fog occurrence. Given that all stations exhibit similar variability (i.e. the timing and magnitude of peaks/troughs in fog occurrence is roughly the same), we focus on the Dutch mean signal, rather than attempting to discern temporal variability at individual stations.

Overall, the interannual variability is characterized by a multi‐year oscillatory signal with a period of approximately 4–6 years (Figure 5). Within each cycle, the observed occurrence of fog can be more than doubled; e.g. in 1958 roughly 9% of the observations were foggy, compared to 1959, when just 4% were. Much of this interannual variability is due to variability in the winter months of December, January, and February. In fact, the wintertime fog anomaly accounts for over 90% of the total annual anomaly (not shown).

**Figure 6:**
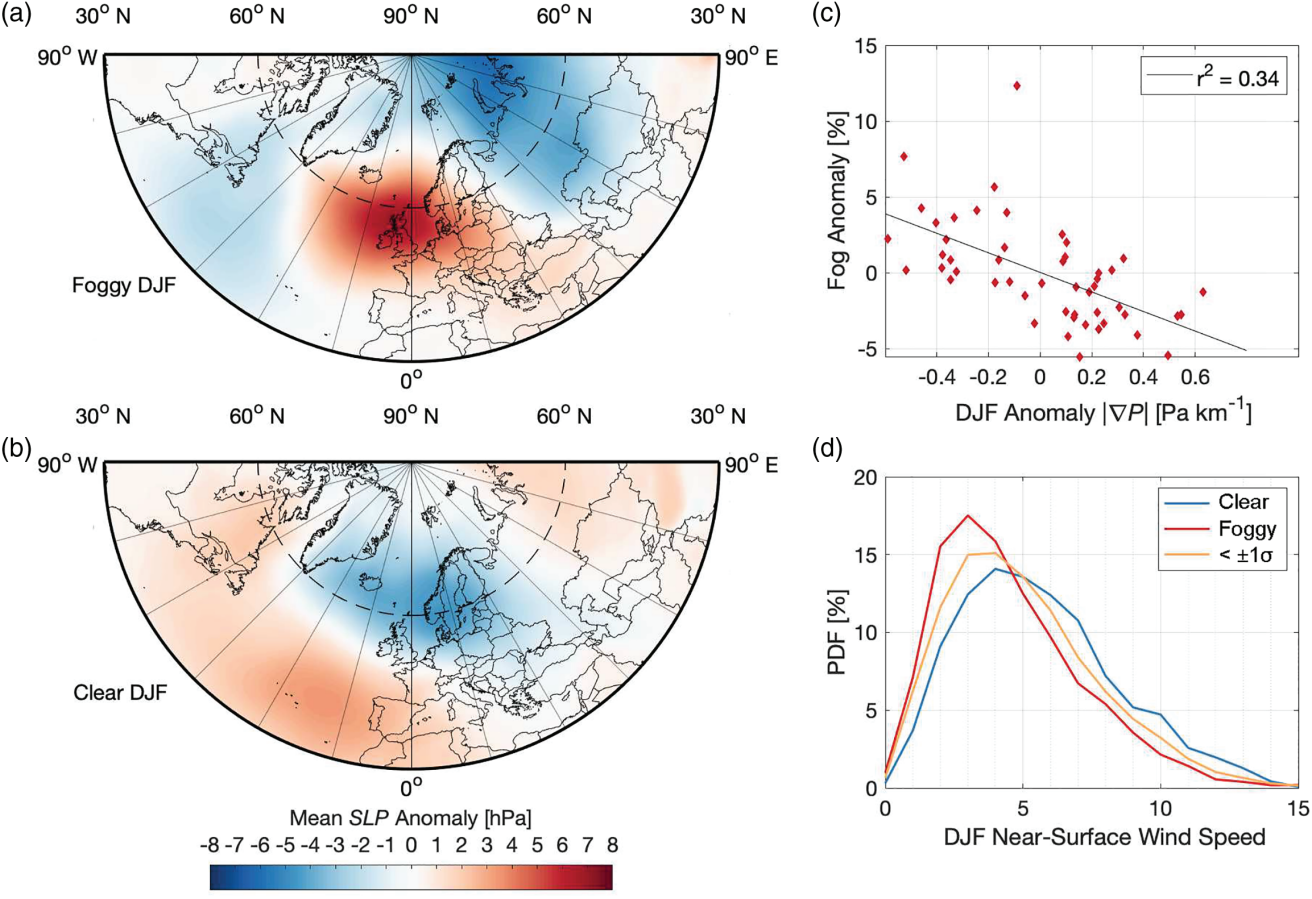
Average winter conditions 1955–2000. (a, b) Mean SLP from the CERA‐20C reanalysis (Laloyaux *et al.* 2018) during years with >1*σ* fog (or clear) anomaly, (c) seasonal fog anomaly as a function of mean wintertime |**∇** *P*|, and (d) PDF of near‐surface wind speeds in foggy and clear winters

In general, positive annual fog anomalies occur in years with winters that have weaker winds, while negative fog anomalies occur in years with winters that experience stronger winds (Figure 6d). Likewise, the frequency of northeasterly winds is higher than average in years with more fog, and lower than average in years with less fog (not shown). This points to the significance of synoptic pressure forcing. We look specifically at years that are either anomalously foggy (foggy years) or anomalously clear (clear years) by more than one standard deviation (1*σ*). In total, there are seven such foggy years, and seven clear years between 1955 and 2000. To assess the synoptic pressure forcing in these years, we make use of the monthly mean sea level pressure (*SLP*) from the CERA‐20C re‐analysis (Laloyaux *et al*. 2018, retrieved at a horizontal resolution of 0.1°). Comparing the mean wintertime (December, January, February) *SLP* anomaly in the foggy years to the mean *SLP* anomaly in the clear years, there is a significant difference in the overall field in the Northern Hemisphere (Figure 6a,b). On average, the mean sea level pressure over Northern Europe is higher in foggy years (a positive anomaly), and lower in clear years (negative anomaly). Important to note is that the increased pressure is not necessarily pointing to high pressure conditions, but rather a weakening of persistent low‐pressure conditions. This is due primarily to the strength and position of the Icelandic low. In foggy years, the 1,000 hPa contour level is shifted further west toward Newfoundland, and reduced in extent, while in clear years, the low‐pressure region covers a much larger area, with lower pressures over Europe.

The change in the wintertime pressure field over Northern Europe leads to changes in the magnitude of the pressure gradient over the Netherlands, |**∇** *P*|. Looking at the wintertime anomalies, there is a negative correlation between the pressure‐gradient forcing and the occurrence of fog (with an r^2^ value of 0.34; Figure 6c); winters that experience weaker forcing (negative anomaly in |**∇** *P*|) are observed to have more fog. Ultimately, the weakened pressure‐gradient forcing is what results in the observed weaker near‐surface wind speeds during foggy years (Van der Linden *et al*. 2017; Figure 6d), which are favourable for fog formation.

Beyond the anomaly in |**∇** *P*|, factors including anomalies in sea surface temperature, the strength of the North Atlantic Oscillation (NAO), the position of the Icelandic Low, and the absolute ***SLP*** anomaly over Europe were all investigated in an effort to identify a single index that could describe the interannual variability in fog occurrence. However, other than the potential link to |**∇** *P*|, no stronger direct correlation could be found linking anomalously foggy/clear years to the other conventional indices. This is not necessarily an indication that there are other synoptic influences at play, but rather that one single index cannot fully describe the relationship between synoptic pressure and fog variability in the Netherlands.

### 3.4 Influences on regional fog occurrence

In this section we look to describe the regional variability of fog in relation to other factors, ideally in terms of non‐meteorological variables. Given the lack of significant topography at most of the stations, elevation is not included. We focus instead on two main factors: the influence of the North Sea, and the role of urbanization.

**Figure 7:**
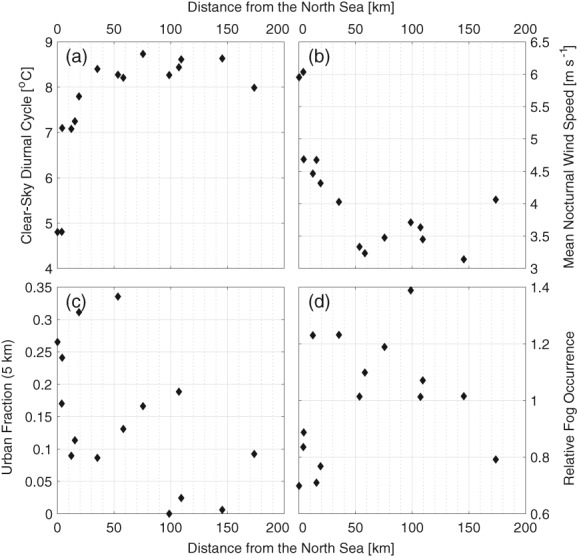
Local properties at the weather stations (1955–2000) as a function of distance from the North Sea. (a) Mean diurnal temperature difference, (b) mean nocturnal wind speed, (c) urban fraction within 5 km, and (d) relative fogginess.

The Dutch climate is strongly influenced by the North Sea. With increasing distance from the coast (i.e. distance from a station location to North Sea, here excluding the IJsselmeer), the magnitude of the clear‐sky diurnal cycle (daytime maximum minus night‐time minimum temperature) increases (Figure 7a). This is due to the high heat capacity of the water, and its modulating effect on local diurnal variability, as well as the fact that wind speeds are, on average, stronger at the coastal stations than inland (Figure 7b). The combination of stronger cooling and weaker winds inland is favourable for fog formation. With distance from the coast, the fraction of fog events that are radiative increases (Figure S2). However, relative fog occurrence is not directly related to distance from the ocean. While the occurrence of fog is indeed lowest at the coast, it is not a monotonic increase inland, with decreased relative fog occurrence further inland (Figure 7d). This nonlinear relation indicates there is more involved than simply an ocean influence.

The coastal region between Rotterdam and Amsterdam is also more urbanized with the presence of the Randstad region. We therefore use the ESA‐CCI land use database (Hollmann *et al*. 2013) to compare the urban fraction; i.e. the fraction of the area surrounding a station (in this case within a radius of 5 km) that is classified as urban, against distance from the ocean (Figure 7c). Indeed, the most urbanized stations are within approximately 50 km of the North Sea, after which they are predominantly rural until Eindhoven (EIN) and Beek (BEE), which are again more urbanized. The general pattern is nearly the inverse of the pattern seen in fog occurrence.

**Figure 8:**
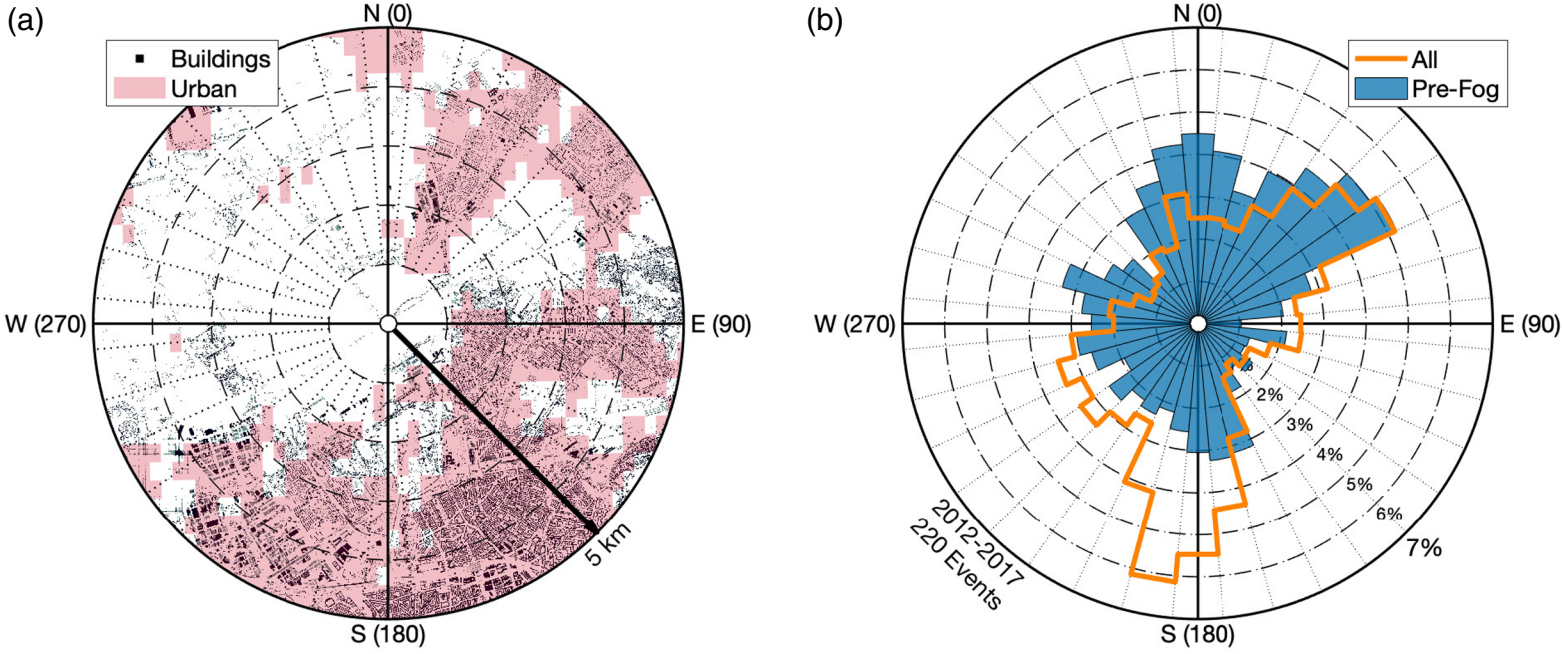
Influence of urbanization on the occurrence of fog at Zestienhoven (ZES). (a) Land use within a 5 km radius of the station with black indicating buildings in the AHN2 elevation dataset, and shading the 300‐m grid cells that are classified as urban in the ESA‐CCI land use database. (b) Probability density function of observed wind directions (i.e. percentage as a function of direction; not a wind rose) for all weak winds with high relative humidity, and the subset that are up to 3 hr before the onset of a radiation fog event

The influence of urban surroundings on fog occurrence is most apparent when looking at the occurrence of fog events at the Zestienhoven weather station (ZES), which is notable for its setting. Located at the Rotterdam–The Hague airport, the city of Rotterdam lies directly to the south, while to the north are predominantly agricultural fields stretching more than 10 km (Figure 8a). The land use contrast can be seen when assessing the wind directions from which radiation fog ultimately forms (Figure 8b). To focus primarily on radiation fog, the observations were filtered according to nocturnal conditions with wind speeds below 5 m/s, relative humidity above 90%, and clear skies. In such cases, the mean wind direction is clearly from the south/southwest (the direction of Rotterdam) and the northeast. If the upwind land‐surface heterogeneity would play no role in influencing the fog climatology, then the distribution of wind direction just before fog events would be expected to have the exact same distribution as the winds in general. However, radiation fog forms almost exclusively when wind is blowing from the north, with a significantly reduced contribution from the south. In other words, radiation fog forms less than half as frequently as would be expected when the wind is blowing from the city of Rotterdam. It should be noted that this distribution does not vary seasonally. While the Zestienhoven station provides the most extreme example of a directional preference for fog formation, directionality is observed at other stations as well (Figure S4), including Schiphol airport (SCH; also a preference for northerlies, with terminal buildings to the southeast), Cabauw (CAB), and Beek (BEE; with increase in fog from the north due to upslope, topographic effects). On the other hand, rural stations, such as Eelde (EEL), show little or no directional preference. It should be noted that wind direction is poorly defined under weak‐wind conditions; however, we assume erroneous observations are normally distributed (i.e. they do not lead to peaks in the distribution).

#### 3.4.1 The regionally weighted index, *RWI*

Based on the apparent ocean and urban influences on Dutch climate and fog occurrence, we identify a simple index combining the two influences of ocean and land use, which we call the “Regionally Weighted Index” (*RWI*). Within a given radius, *R*, the fraction of a given angular bin (i.e. wedge) that is classified as either urban or ocean (*f*_uo_) is determined (here, from the ESA‐CCI land‐use database; Hollmann *et al*. 2013). This “urban or ocean fraction” is calculated as simply the number of grid points within a wedge that are classified as either urban or ocean, divided by the total number of gridpoints within that wedge. The weighted mean of all angular bins (where *a* refers to the bin index) is then taken as

(2) $$\text{RWI}\left(R\right)=\underset{a}{\sum }w\left(a\right)f_{\text{uo}}\left(a,R\right).$$

The weights, $w\left(a\right)$, are calculated from the underlying wind distribution (e.g. PDF in Figure 8b). This allows more weight to be given to the region from which the wind predominantly blows. In the case where the wind PDF is not known, the *unweightedRWI* is simply the mean value of $f_{\text{uo}}\left(a,R\right)$ (i.e. *w*(*a*) is equal to 1/*n*_bins_, where *n*_bins_ is the number of wedges). It should be noted that the use of the index to compare the relative likelihood of fog at two different locations requires that they are in similar settings (for example, that the synoptic climatology is the same, as well as aerosol quantity and composition).

As a simple example, assume a region divided into four quadrants. In each of the four angular bins, *f*_uo_ is 0.75, 0.5, 0.25, and 0, respectively. The unweighted value of *RWI* is the mean: 0.375. If the wind blows 70% of the time from quadrant 1, and equally from the others (10%), then *w* is 0.7, 0.1, 0.1, and 0.1, respectively, and the wind‐weighted *RWI* is equal to 0.6. On the other hand, if the wind blows 70% of the time from quadrant 4, and equally from the others (10%), then *w* = 0.1,0.1,0.1, and 0.7, respectively, and *RWI* is just 0.15.

**Figure 9:**
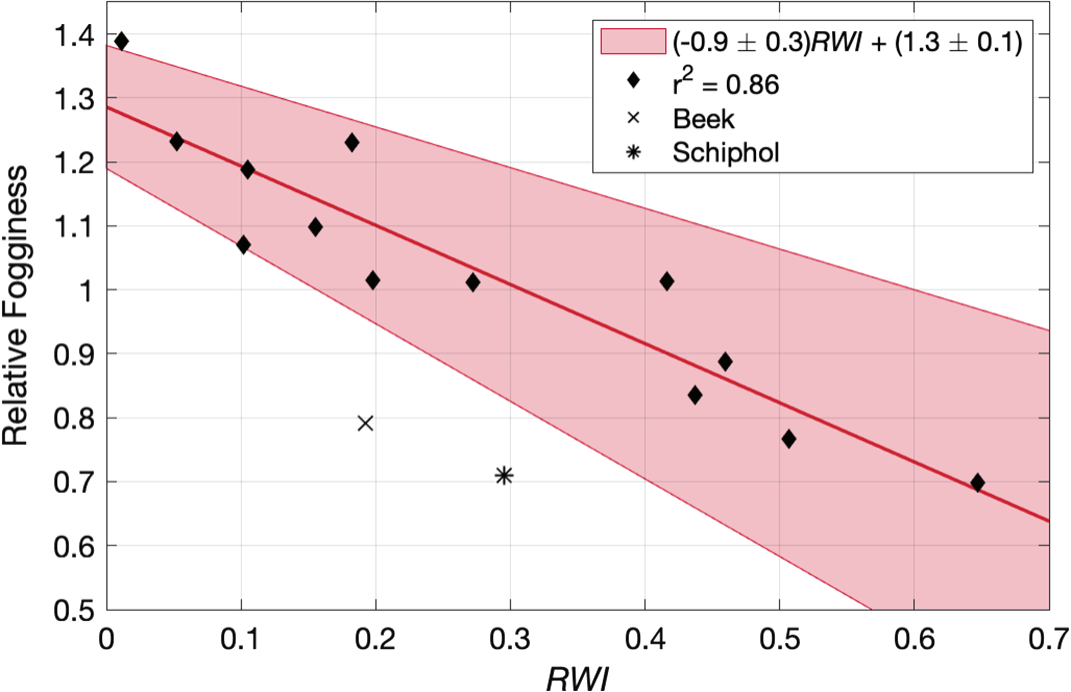
Relative fogginess as a function of *RWI* (Equation 2) calculated using 36 angular bins and a radius of 5 km. The linear regression excluding Beek (BEE) and Schiphol (SCH) is shown with 95% confidence intervals indicated by the shading (Equation 3) [Colour figure can be viewed at wileyonlinelibrary.com]

Here we calculate *RWI* at each station in the long‐term dataset using 36 angular bins (centred every 10°) and a radius of 5 km. This radius was chosen in order to allow for sufficient data points (e.g. 1 km would include only a limited number of land‐use cells given the 300‐m resolution of the database), while at the same time ensuring that the index is still regional (a radius of 10 km would include locations too far away from the observation site to be relevant). The calculated values range from as low as 0.05 at the rural station of Deelen (DEE), up to 0.65 at the coastal‐urban station of Vlissingen (VLS). Figure 9 shows the comparison between relative fogginess and *RWI* at all stations, weighted according to the underlying wind PDF at each station (as in, e.g., Figure 8). An analogous figure showing the relationship between *RF* amd the unweighted *RWI* is presented in Figure S5. While not perfect, the agreement between the two variables is striking, with correlations of 0.56 and 0.62 for the unweighted and weighted indices, respectively. Only two stations deviate significantly from the others, Beek (BEE) and Schiphol (SCH). This is not surprising. Beek, as above, is in the most complex terrain of all stations, and is meteorologically isolated from the other stations, with few fog events occurring at the same time as at other stations. Being located at a major international airport, the Schiphol station is surrounded by far more concrete and urban construction than is resolved in the land‐use dataset (i.e. should be further to the right on the *x*‐axis). Further, the constant flight activities of the airport can have a significant impact on the localized meteorology (Appendix). The two stations are also statistical outliers according to the Generalized Extreme Studentized Deviate test (GESD; Rosner, 1983). Removing the two stations from the regression for the physical reasons mentioned above, the strength of the correlation increases significantly, up to 0.82 and 0.86 for the unweighted and weighted calculations, respectively, with *RWI* providing a strong indicator of whether or not one location may be regionally favourable for fog occurrence compared to another.

With Beek and Schiphol excluded, the regression (and 95% confidence intervals) relating relative fogginess, *RF*, to *RWI* is

(3) $$RF=\left(-0.93\pm 0.29\right)\text{RWI}+\left(1.3\pm 0.1\right).$$

If using the unweighted *RWI*, the slope and intercept are −0.85 ± 0.27 and −1.3 ± 0.1, respectively.

## 4 DISCUSSION

Fog occurrence in the Netherlands over the past half century was assessed using observations from a dense network of stations. In spite of the relatively small extent and generally uniform topography of the Netherlands, the overall occurrence of fog was shown to vary significantly, even within a few kilometres. In general, stations in the centre and northeast of the country exhibit greater fog occurrence than those in the south, and particularly those near the coast (Figure 3).

Since the mid‐1950s, fog occurrence has decreased – on average – throughout the country (Figure 5). Boers *et al*. (2015) attribute much of this decline to the changing quantity and composition of aerosols. However, the trend is not uniform throughout the country, with the largest decrease occurring at Eindhoven (EIN); a 50‐year reduction of over 70% of the original value (–0.11% per year). On the other hand, the De Kooy station (DeK) showed comparatively little change in observed fog occurrence over the same period. We do not investigate the causes for this in great detail; however, it is likely related – at least in part – to the relative changes in urbanization over the past few decades. Eindhoven experienced a technology boom in the second half of the 20th century, with the founding of its Technical University, and the expansion of the Phillips electronic company, with significant population growth over the same period (Ekamper *et al*. 2003). Of course, linking the socio‐economic expansion of a region to fog occurrence is tenuous, but not unreasonable.

While the general trend in fog occurrence is negative throughout the past half century, the interannual variability of fog occurrence is much greater, accounting for over a factor of 2 difference from one year to the next (Figure 5). No strong quantitative relationship could be found to relate the fog anomaly to synoptic indices (e.g. the NAO); however, there is a clear signal in the large‐scale pressure‐gradient forcing over Europe in years with significantly more or less fog (Figure 6). Particularly in anomalously foggy years, the wintertime sea level pressure was higher, on average, over northwestern Europe, corresponding with a westward shift of the Icelandic Low toward Newfoundland, and a general weakening of low‐pressure conditions over northern Europe. The result of the *SLP* anomaly is primarily manifest as a change in wind speed, due to a change in pressure‐gradient forcing. Van der Linden *et al*. (2017) showed that different boundary‐layer stability regimes can be classified according to the pressure‐gradient forcing, with weaker forcing corresponding to more stable nocturnal boundary‐layer conditions. As such, one can infer from the weaker pressure gradient that the nocturnal conditions are more often (very) stable in the foggy years. Given that stable conditions are favourable for the formation of radiation fog, and that radiation fog is the most common type of fog in the Netherlands, this is significant.

Fog as far away from the North Atlantic as the Indo‐ Gangetic plains has been linked to Northern Hemisphere teleconnections (Hingmire *et al*. 2019). However, while we could find no such direct link to a single teleconnection, the link between large‐scale pressure forcing and Dutch fog that we find is not surprising, given the established relationship between synoptic pressure fields, including the state of pressure‐defined teleconnections, and weather in Northwestern Europe. For example, previous studies have shown links between interannual variability in Northwestern European temperature and wind speed – particularly in winter – to such synoptic influences as the North Atlantic Oscillation, and even the ENSO (Toniazzo and Scaife, 2006; Hirschi and Sinha, 2007; Riaz *et al*. 2017; King *et al*. 2018; KNMI, 2019). It should be further noted that, while we only claim weak correlation between the pressure‐gradient forcing and the fog anomaly, the other studies also found only relatively weak correlations (∼0.4) between the synoptic indices and their relevant variables of interest. In terms of predictability, it is conceivable that the large‐scale pressure gradient might be used to forecast in advance whether a given winter may be more or less foggy than usual, even though a direct quantitative relationship is difficult to define. However, the exact the pressure field, including the state of such teleconnections as the NAO – particularly the onset of anomalous events – are difficult to forecast beyond a few days to weeks (e.g. Jung *et al*. 2011; Domeisen *et al*. 2018). As such, the the utility of such forecasts for statistical fog prediction (i.e. being able to say whether a given winter will be more or less foggy than average) is limited.

Regionally, we relate the relative occurrence of fog to the mesoscale surroundings of a station. Specifically, stations that are in a more urban‐ or ocean‐influenced environment are observed to have less fog on average than those in more rural, inland settings (e.g. Figure 3). This is due to the thermal and climatic influence of the surfaces, through, for example, the influence of the urban heat island effect (e.g. Bendix, 1994; Sachweh and Koepke, 1995, 1997) and the increased thermal capacity of the water. A striking example of the role surrounding conditions play in the relative occurrence of fog is the Zestienhoven station where, in direct contrast to the underlying wind distribution, fog rarely forms when wind is blowing from the city of Rotterdam (Figure 8). The results are similar to those found by Tardif and Rasmussen (2007), with urbanization significantly reducing the overall fog occurrence. However, they also found increased fog occurrence at coastal stations, whereas the most fog was observed in the centre of the Netherlands. This could be due to the difference in landscape (more complex terrain in the New York City study region), or climatological differences in, for example, offshore water temperatures and prevailing wind direction.

Our analysis does not look directly at where the observed fog is formed. However, it is possible that fog may form in one location, and then be advected elsewhere (for example, inland fog advected to the coast, or sea fog advected inland, by the land–sea circulation). That being said, systematic advection of fog is still a regional effect that would naturally be included in the analysis. At the same time, if it is not occurring systematically but randomly, then it will also not affect our climatological analysis as random events will be masked by more dominant patterns.

One factor we did not – and could not – consider in detail is the role of aerosols in determining the relative occurrence of fog. This was primarily due to the complexity of assessing the role aerosols play in terms of both hygroscopicity and overall number concentrations, as well as the limited availability of coincident observational data. However, one would expect that aerosol composition is highly variable throughout the country, influenced by such factors as upwind urbanization or agriculture. At the same time, the ocean influence extends to the aerosols, with sea salt (a hygroscopic cloud condensation nucleus, CCN) most abundant near the coast and decreasing in concentration with distance from the ocean. Manders *et al*. (2009) show that the decrease is nearly linear from the ocean toward the southeast, with the lowest aerosol concentrations in the country found near Beek (BEE). This may, in part, be an explanation for the low occurrence of fog at Beek. However, a full aerosol study would be required to assess the overall impact on fog throughout the country.

### 4.1 Applying *RWI*

**Figure 10:**
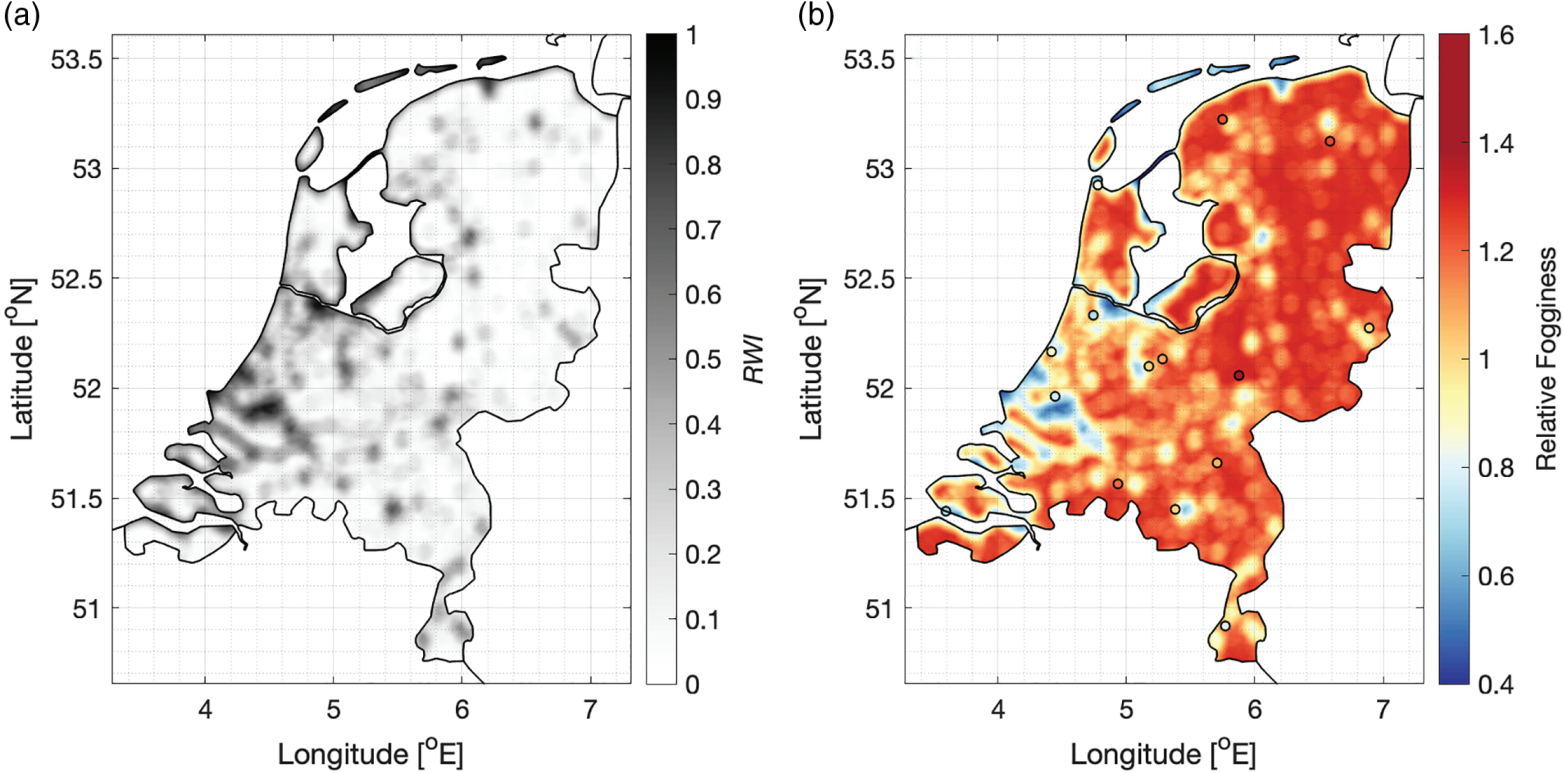
Map of the Netherlands showing the (a) unweighted *RWI* calculated from the ESA‐CCI Land Use database, and (b) the relative fogginess (from Equation 3) expected throughout the country. The observed relative fogginess at the weather stations is shown in the colour of the station points, allowing comparison with the estimated value

The concept of the Regionally Weighted Index (*RWI*; Equation 2) demonstrates that knowing the surrounding land use leads to the ability to determine – on the mesoscale – whether one location will have more fog than another (assuming the stations are in similar settings; e.g. the synoptic conditions are the same, as well as the aerosol content). Figure 10, for example, shows the unweighted *RWI* (i.e. without observations of wind directon) in the Netherlands converted to a relative fogginess map. Coastal and urban influences are immediately apparent. While for a vastly different region, the hypothetical map is similar in character to the image in Lee (1987), with urban “pockets” clearly discernible. *RWI* can, as a result, potentially be used with a view toward practical applications. Unlike existing indices, such as the fog potential index of Perry and Symons (2002), *RWI* is straightforward to define, relying on quantitative measures, without subjective attribution of a value to a given variable (e.g. the “general expression of any environmental factors”).

Unfortunately, the stations investigated here are still not as diverse as would be desired. While the mesoscale variability is large, *in situ* characteristics of the stations are largely similar. For instance, given that all stations assessed conform to WMO standards, they are all locally above grass. This means that highly localized features, such as the immediate land cover or soil type cannot be easily assessed, in spite of their impact on near‐surface temperature inversions (van de Wiel *et al*. 2017). Likewise, while the flat terrain of the Netherlands allows for simpler analysis of other factors, regions with more complex topography will need to be considered, perhaps including an additional scaling term that measures the variability of topography, such as the variance, or even the divergence (−∇^2^) which could give an indication of where cold air would be likely to pool. Further testing of *RWI* in a range of settings – through further observational and numerical studies – is therefore required, and an additional scaling may need to be incorporated. It should also be noted that the value of *RWI* changes with *R* (the radius of interest), and the land‐use dataset from which the urban/ocean fraction, *f*_uo_, is calculated. While a radius of 5 km was chosen in order to balance representativity with sufficient land‐use data, the “correct” value for *R* is difficult to define objectively. Perhaps a weighted distance can be included to give more weight to closer cells, making the choice of *R* insignificant.

The two outliers in the relationship between relative fogginess and *RWI* – Beek (BEE) and Schiphol (SCH) – are obvious outliers in terms of the stations' physical characteristics. Beek, located in the south of the country, is completely isolated from the other stations with an independent fog climatology, and surrounded by complex topography. It may also have lower CCN concentrations in the form of sea salt aerosols (Manders *et al*. 2009). This violates the assumption that the stations be in a similar synoptic setting. Likewise, the Schiphol station is located in the highly urbanized setting of one of Europe's busiest airports. The buildings and runways are not resolved in the wider land‐use analysis, nor can airport operations be accounted for, such as the movement of aircraft, which can increase surface temperatures significantly (Appendix), pointing to a “built‐in” fog dispersal system.

## 5 CONCLUSIONS

The Netherlands provides an excellent setting for studying the influences on fog occurrence in the absence of significant topography. Through the long‐term analysis of visibility observations throughout the country, fog is shown to be highly variable in both time and space. Interannual variability in the observed signal is shown to be related to changes in the synoptic pressure field over the Northern Hemisphere, with increased wintertime sea level pressure over Scandinavia and northwestern Europe leading to increased fog occurrence – likely related to the increased stability of the near‐surface boundary layer. This interannual variability is considerably larger than the observed long‐term decrease in fog.

Interstation variability is similarly large throughout the Netherlands. Over the past 45 years, fog was observed up to twice as frequently in rural locations as in semi‐urban and coastal locations. Combining this, a simple index was identified to describe the mescoscale influences of water bodies and urbanization, providing an indication of whether one location will have (relatively) more fog than another. This has very practical applications, with the potential to assist, for example, in infrastructure planning and or risk assessments (even without the need for long‐term meteorological observations when using the unweighted *RWI* as in Figure 9). However, it will first need to undergo further extensive testing in a range of settings, such as over different land surfaces.

We suggest *RWI* also be used in other locations beyond the Netherlands, testing its limitations and potential. Provided the separation between locations is not too large – and therefore synoptic setting is similar – it should be able to provide a consistent estimate of the relative fogginess between two locations. While complex topography will affect the comparison, it may be possible to add another weighted term describing topographic variability in the case where two locations are not the same; for example, the elevation variance, or the relative elevation of the location to its surroundings. In order to facilitate the analysis, satellite‐derived climatologies, such as presented by Egli *et al*. (2019) would be extremely useful.

The observational results presented here also have wider implications for the simulation of fog. They further highlight the importance of the various range of scales on which fog is influenced. The climatology of fog is driven by wider mesoscale and synoptic forcing conditions. Particular attention should therefore be paid to ensuring the accuracy of synoptic forcing and mesoscale land surface characteristics. That being said, individual fog events, as opposed to the climatology, will be highly sensitive to localized conditions. This reinforces the need to have accurate models on a range of scales, which has been identified in several previous works (e.g. Gultepe *et al*. 2007; Steeneveld *et al*. 2015).

## APPENDIX: WEATHER MODIFICATION BY AIRCRAFT

Fog occurrence is shown to be considerably lower than would be expected at Amsterdam's Schiphol International Airport (station SCH here) when compared to other stations in the Netherlands. This is likely related to the unique environment around Schiphol, and indeed most major airports.

**Figure A1:**
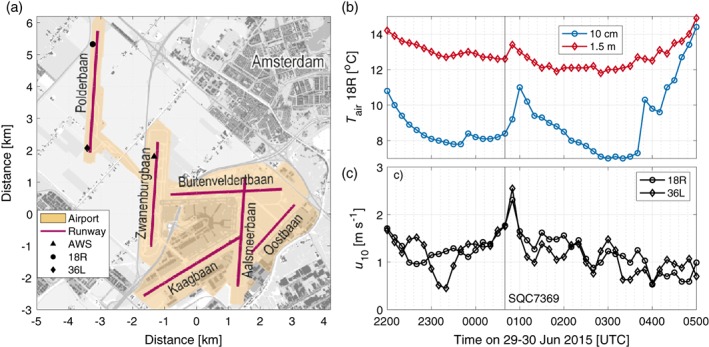
(a) Map of Schiphol airport and surroundings (modified from: CLO, 2017). Ten‐minute averaged observations of (b) 10 cm and 1.5 m temperature next to runway 18R, and (c) 10 m wind speed measured next to 36L and 18R, with the northbound take‐off time of flight SQC7369 indicated – the only large aircraft to take off during the night – just after 0040 UTC. [Colour figure can be viewed at wileyonlinelibrary.com]

According to the ESA‐CCI land use database, the majority of the area surrounding Schiphol is agricultural. However, it is locally highly urbanized, with the expansive terminal buildings and large areas of concrete making up the many taxiways and runways (Figure A1). Bergot *et al*. (2015) show that heterogeneous surfaces and airport structures can have a large impact on localized fog formation. At the same time, the airport is spread over a large area (close to 3000 hectares; Schiphol, 2019), with the most remote Polderbaan runway (18R/36L) located more than 4 km from the main terminal buildings. Observations show that there is a significant difference in fog occurrence measured around the airport, with the Polderbaan far more susceptible to fog and low‐visibility conditions (Kattenberg *et al*. 2013).

There is also a further highly localized feature at Schiphol (in both space and time) that affects the observed weather: aircraft. Schiphol is one of Europe's busiest airports, with almost 500,000 aircraft movements (take‐off/landing) per year (Schiphol, 2019). First shown in Schulte (2017), the take‐off of aircraft can lead to temporarily elevated near‐surface wind speeds, and subsequent near‐surface warming.

Figures A1b,c show 10‐min averaged temperature and wind speed measurements made at stations located along the Polderbaan and Zwanenburgbaan (18C; the AWS used in this study) runways on the night of 29–30 June 2015. Skies remained cloud‐free throughout the night, which, combined with weak wind conditions, led to very stable temperature stratification near the surface (a maximum difference of 4.5 °C between 10 cm and 1.5 m). A few double‐engine aircraft (e.g. Airbus 321) took off on runway 36L (northbound take‐off on the Polderbaan runway) during the night, but only one very large aircraft, a Boeing 747 with four engines (Singapore Airlines Cargo flight 7369). The observations show a spike in wind speed, followed by near‐surface warming at the same time as the flight's northbound departure on the Polderbaan runway. At 10 cm height, the increase in temperature is around 3 °C! This is most likely due to both the downward heat transport from the engine wake (through enhanced turbulent mixing and vertical transport), and the input of energy to the system from engine exhaust. After take‐off, it took an hour for temperature to recover to pre‐take‐off conditions. This is one of the most extreme examples in the dataset, but it is not the only example.

The near‐surface warming caused by jet engines has implications for fog at airports. Appleman and Coons (1970) showed that the wake of a stationary jet can dissipate fog in a matter of minutes, in a fashion similar to the Fog Investigation Dispersal Operation (FIDO) system developed during the Second World War, which used flames on either side of the runway to mix and evaporate thick fog so that aircraft could land (Popular Science, 1945). Combined with the thermal influence of the urbanized surfaces at airports, the increased turbulent mixing (including downward mixing of drier air aloft), and reduced cooling or periods of sudden warming due to aircraft operations, will make the sites unfavourable for fog, particularly radiation fog. That is likely why Schiphol appears to have less fog than would be expected when compared to other Dutch stations (Figure 9).

Overall, the highly local influence of airport urbanization and aircraft movements may result in a surprising benefit. While fog can have a large impact on airport operations, they ultimately have “built‐in” fog mitigation. The benefit grows with the size of the airport. The larger and busier the airport (e.g. Heathrow and Paris–Charles de Gaulle), the more damaging a fog event can be, but there is greater potential for disruption of fog formation due to air traffic. While analysis is naturally limited here, the “airport effect” on nocturnal weather would be extremely interesting for future study, both observationally and numerically, particularly the impact of aircraft on fog formation/dissipation.

## Supporting information


**Figure S1**. Monthly mean temperature and fog occurrence at the stations in the long‐term analysis, compared to the North Sea surface temperature.
**Figure S2**. Relative occurrence of radiation fog at the short‐term stations (analogous to Figure 3).
**Figure S3**. Probability density functions (PDFs) of the time of onset, duration, and mean visibility of observed fog events at the short‐term stations.
**Figure S4**. Observed wind directions preceding radiation fog for all short‐term stations at which cloud data are available (analogous to Figure 8b).
**Figure S5**. Relative fogginess as a function of the unweighted *RWI*, analogous to Figure 9.
