# Supplementary material for: Dutch fog: On the observed spatio‐temporal variability of fog in the Netherlands
Source: Q J R Meteorol Soc. 2019 Aug 2;145(723):2817–34. doi: 10.1002/qj.3597 (PMC6771507; doi:10.1002/qj.3597)
Supplement: Supplementary file 1 — Figure S1. Monthly mean temperature and fog occurrence at the stations in the long‐term analysis, compared to the North Sea surface temperature. Figure S2. Relative occurrence of radiation fog at the short‐term stations (analogous to Figure 3). Figure S3. Probability density functions (PDFs) of the time of onset, duration, and mean visibility of observed fog events at the short‐term stations. Figure S4. Observed wind directions preceding radiation fog for all short‐term stations at which cloud data are available (analogous to Figure 8b). Figure S5. Relative fogginess as a function of the unweighted RWI, analogous to Figure 9. [file QJ-145-2817-s001.pdf]

SUPPORTING FIGURES

Dutch Fog: On the Observed Spatio-Temporal Variability of Fog in the Netherlands

Jonathan G. Izett[1] | Bas J. H. van de Wiel[1] | Peter Baas[1] | J. Antoon van Hooft[1] | Ruben B. Schulte[2,3]

[1]Department of Geoscience and Remote Sensing, Delft University of Technology, Delft, The Netherlands

[2]Meteorology and Air Quality Group, Wageningen University and Research, Wageningen, The Netherlands

[3]Netherlands National Institute for Public Health and the Environment (RIVM), Bilthoven, The Netherlands

Correspondence
Stevinweg 1, 2628 CN, Delft, The Netherlands
Email: j.g.izett@tudelft.nl

Funding information
European Research Council Consolidator Grant: 648666

This document presents supplementary figures for the article entitled "Dutch Fog: On the Observed Spatio-Temporal Variability of Fog in the Netherlands" by Izett et al. (2019). Included are five figures, Figures S1–S5, respectively.

KEYWORDS
Fog, Climatology, Regional Variability, Weather, Land Use

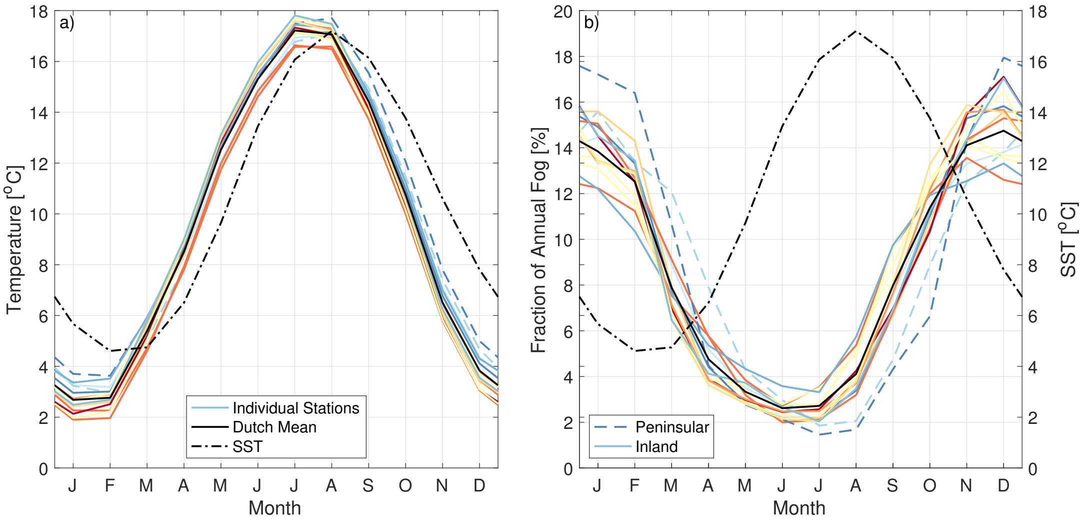

FIGURE S1 Mean monthly a) air temperature, and b) fraction of annual fog occurrence compared to mean observed sea surface temperature (SST) of the North Sea for all stations in the long-term analysis from 1955–2000. The mean of all stations is shown in solid black, while the dashed lines distinguish the peninsular stations of Vlissingen (VLS) and De Kooy (DeK) from the inland stations (solid). The colours correspond to those used in Figure 3 of the article (red=more fog).

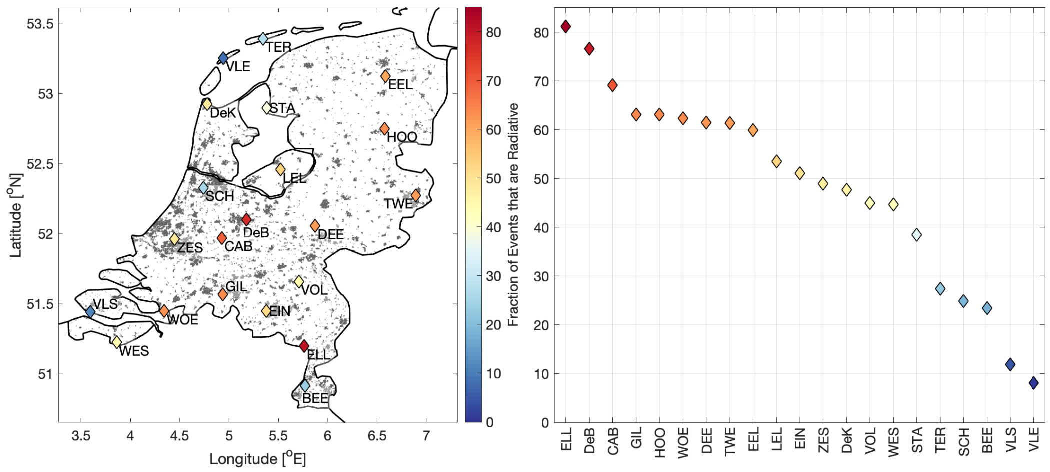

FIGURE S2 Fraction of fog events at each station between 2012 and 2016 that are classified as radiation fog according to the methodology in Section 2.2.

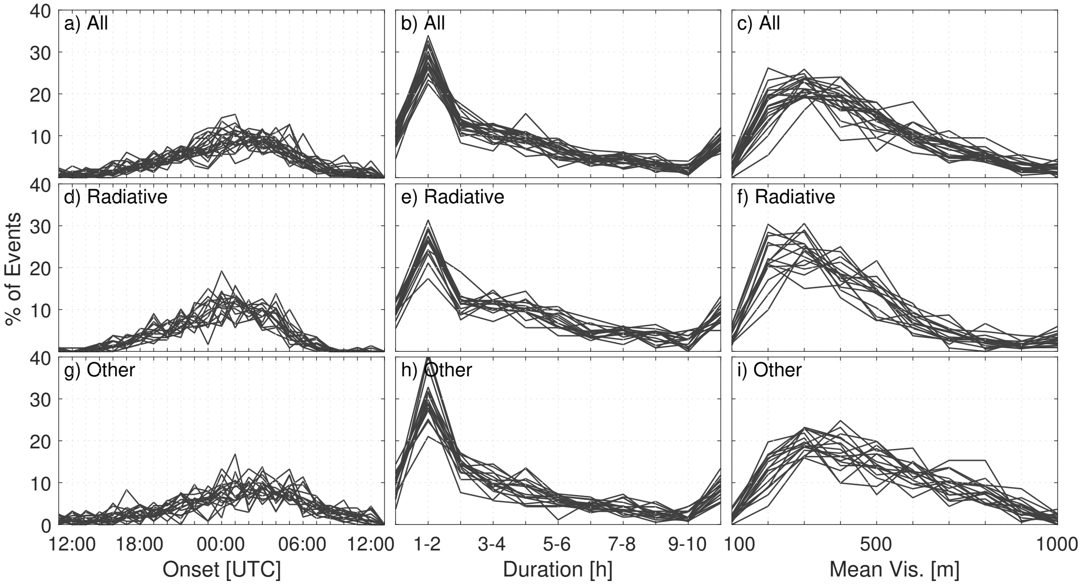

FIGURE S3 Probability density functions (PDFs) showing the characteristics of fog events for all stations in the short-term analysis (2012-2016). adg) time of onset, beh) duration, and cfi) mean visibility for, a-c) all fog events, d-f) the subset of events that are classified as radiative fog events, and g-i) events that are other fog types. The simple algorithm presented in Section 2.2 was used to classify events.

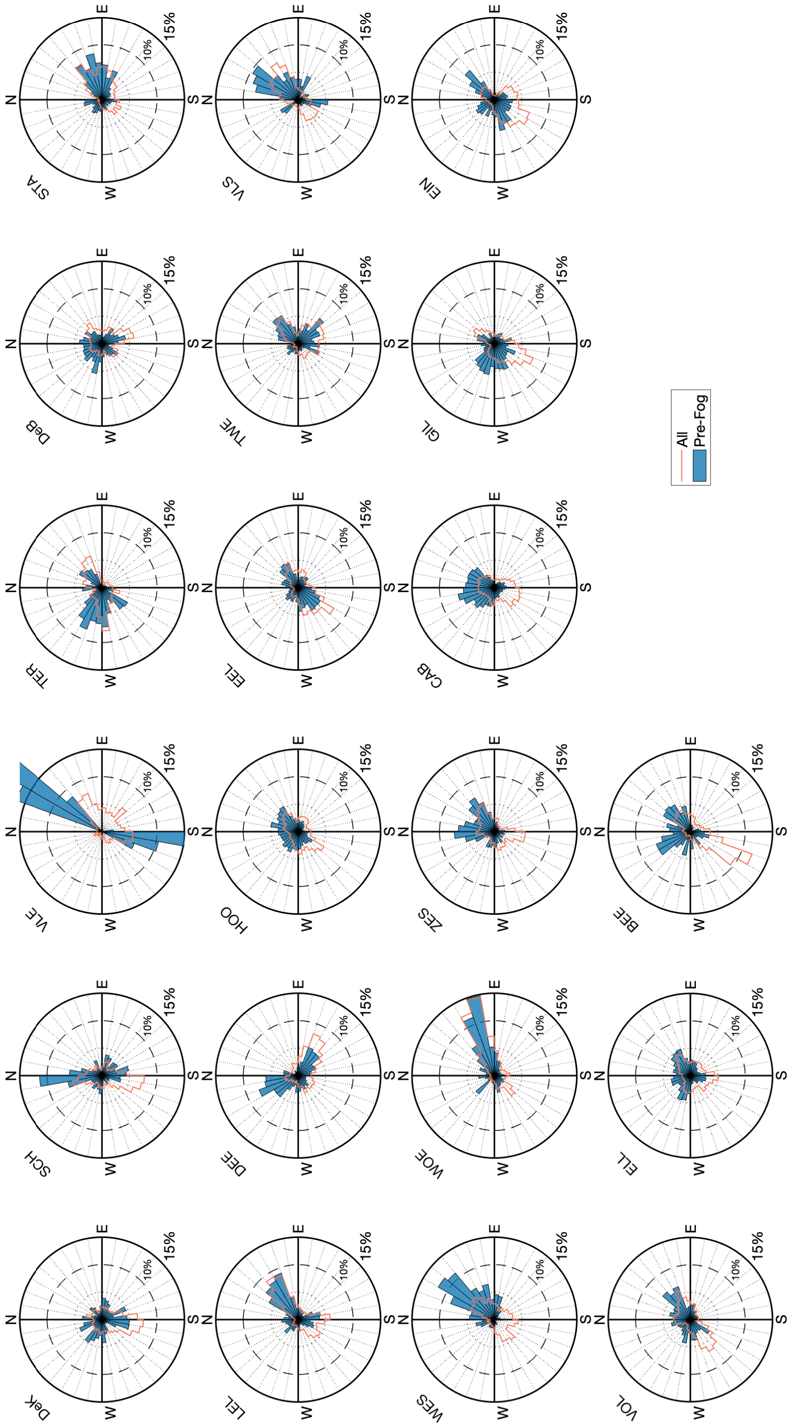

FIGURE S4 PDFs of observed clear-sky nocturnal wind direction for all stations when wind speed is $< 5\,\mathrm{m\,s^{-1}}$ and relative humidity is $> 90\%$, including the subset that are up to 3 hours before the onset of a radiation fog event as classified by the simple algorithm in Section 2.2. (Analogous to Figure 8b in the main document).

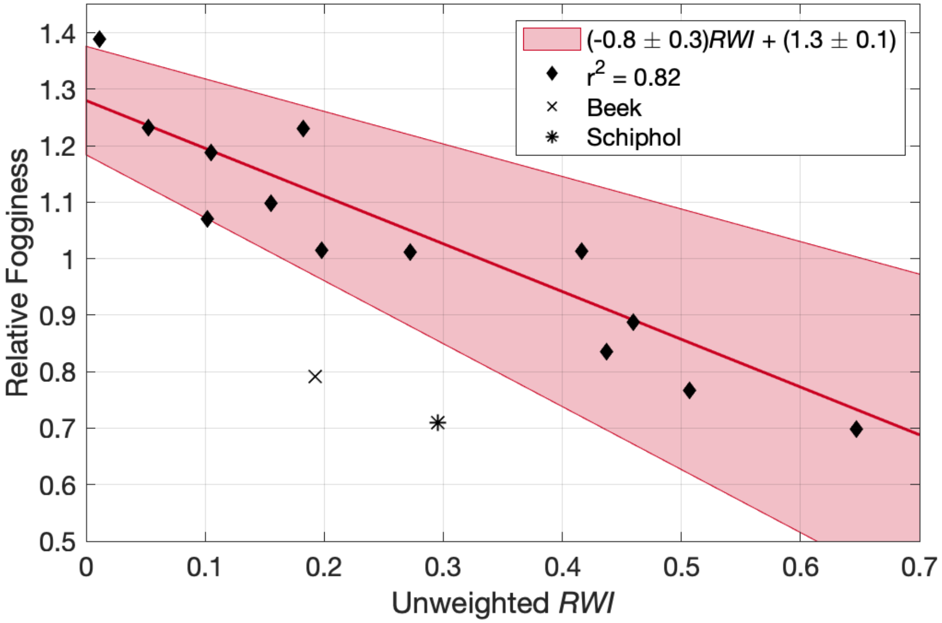

FIGURE S5 Relative fogginess as a function of *unweighted RWI* (Equation 2) calculated using 36 angular bins and a radius of 5 km. The linear regression excluding Beek (BEE) and Schiphol (SCH) is shown with 95% confidence intervals indicated by the shading.
